# Supplementary material for: The frequency of workplace violence against healthcare workers and affecting factors
Source: PLoS One. 2023 Jul 28;18(7):e0289363. doi: 10.1371/journal.pone.0289363 (PMC10381052; doi:10.1371/journal.pone.0289363)
Supplement: Attachment — Submitted filename: Response to Reviwers.docx [file pone.0289363.s001.docx]

03-April-2023

**Dear Editor,**

Respond to the reviewers’ comments for the manuscript ID PONE-D-23-03576 entitled **"The frequency of workplace violence against healthcare workers and affecting factors"**

First of all, we would like to thank you, your editorial staff, and the expert reviewers for your very valuable comments, feedback, and suggestions. During our review of your changes and suggestions, we paid close attention to each one. Below are our responses to the changes and suggestions. It is our hope that the changes we have made to the redressed version of our manuscript will be acceptable for publication in your respected journal after the revisions we have made.

Sincerely yours,

Hıdır SARI, MD.

**Response to Additional Editor Comments:**

1. The methodology should be expanded. It does not give the essence of what you have actually conducted. (eg: Were the 135 participants questioned or was it only the records that were used?)

**Response 1**: By extending the methodology, relevant changes to the paper are marked in yellow

2. The funding sources should be declared. If no funding received, please indicate that.

**Response 2:** “The authors received no specific funding for this work.” sentence was added in Declaration section of manuscript.

3. The recommendations seems to be not arising from your findings. Please give recommendations based on your original work.

**Response 3:** Recommendations in line with the study findings were made and added to the conclusion section.

[Note: HTML markup is below. Please do not edit.]

**Response to Reviewers' comments:**

Reviewer's Responses to Questions

Comments to the Author

1. Is the manuscript technically sound, and do the data support the conclusions?

The manuscript must describe a technically sound piece of scientific research with data that supports the conclusions. Experiments must have been conducted rigorously, with appropriate controls, replication, and sample sizes. The conclusions must be drawn appropriately based on the data presented.

Reviewer #1: Partly

Reviewer #2: Yes

2. Has the statistical analysis been performed appropriately and rigorously?

Reviewer #1: N/A

Reviewer #2: Yes

3. Have the authors made all data underlying the findings in their manuscript fully available?

The PLOS Data policy requires authors to make all data underlying the findings described in their manuscript fully available without restriction, with rare exception (please refer to the Data Availability Statement in the manuscript PDF file). The data should be provided as part of the manuscript or its supporting information, or deposited to a public repository. For example, in addition to summary statistics, the data points behind means, medians and variance measures should be available. If there are restrictions on publicly sharing data—e.g. participant privacy or use of data from a third party—those must be specified.

Reviewer #1: Yes

Reviewer #2: Yes

4. Is the manuscript presented in an intelligible fashion and written in standard English?

PLOS ONE does not copyedit accepted manuscripts, so the language in submitted articles must be clear, correct, and unambiguous. Any typographical or grammatical errors should be corrected at revision, so please note any specific errors here.

Reviewer #1: Yes

Reviewer #2: Yes

5. Review Comments to the Author

Reviewer #1: Thanks for the opportunity to review the manuscript titled “The frequency of workplace violence against healthcare workers and affecting factors”. The manuscript addresses an important and relevant issue regarding e the influencing factors of workplace violence (WPV) events and their legal processing, as well as the frequency of WPV against HCWs. The authors performed an observational, descriptive, retrospective frequency study. The objectives are stated clearly, but methods are insufficient and unclear. The small sample size (n. 135 healthcare workers) does not allow to draw significant conclusions. Therefore, the aforementioned limitation does not allow to attribute significance to the results of the study. The authors did not assess the way to minimize such limitation of the study.

**Response to Reviewer #1:** The study was conducted retrospectively through hospital records, so all violence records of the Patient Rights and Employee Safety and Law departments were analyzed. There were only 135 reports of violence among healthcare workers. This was described in the method section

Reviewer #2: Thank you for the opportunity to review this article. The topic is certainly interesting and relevant. Some minor changes are needed before the article can be published.

Much of the article is well written. I am not sure why the authors have included Table 1 as it does not provide relevant information. It is also not clear if the statistics in Table 1 are related to the hospital that they are researching or to hospitals in general.

**Response to Reviewer #2:** Table 1 was removed from article.

In the introduction, they refer twice to workplace violence happening to ‘important people’ – I am not sure who they mean by this, and it would be good to clarify.

**Response to Reviewer #2:** Occupational accident and violence definition was used in the introduction of the manuscript according to a report published by the International Labor Organization (ILO). The relevant definition sentence has been reorganized in the introduction.

In the ‘Legal Process’ section, it would be good to have a few sentences at the start explaining the section.

**Response to Reviewer #2:** ‘’According to Turkish legal system, legal processes and definitions of violence against health workers reported with white code application.’’ sentence was added.

In the abstract and in the results section after you mention 1.2%, you have a bracket ‘)’ that needs to be removed.

**Response to Reviewer #2:** Have been corrected.

The authors stated that they organised the data within the categories of ‘non compliance with procedure, communication and dissatisfaction’. It would be good to clarify how and why they came up with these 3 headings as it seems that they were pre-selected by the authors.

**Response to Reviewer #2:** These definitions were added and described in the method section.

In terms of the findings, more clarification is needed as to why 135 healthcare workers were included in the study, but then 10821 healthcare workers were reported as the cumulative total. Is it that there were 10821 incidences? Please clarify this.

**Response to Reviewer #2:** This clarification was added and described in the method section.

If it is only 135 health care workers, then there are l**ikely to be healthcare workers with multiple experiences of violence** and this really needs to be discussed and acknowledged.

**Response to Reviewer #2: ‘’**Any HCWs with more than one experience of violence were not found in the violence records’’.

In the discussion section, in the paragraph where you begin with ‘In a tertiary hospital, 345 cases of violence were examined based on white code reports…’, are you talking about the study’s hospital or another hospital?

**Response to Reviewer #2:’’** Polat and Çırak conducted at a tertiary hospital , 345 cases of violence were examined based on white code reports. ‘’ This sentences added in the discussion section.

In the discussion it would also be good to talk about the influence of culture on workplace violence as your study is specific to Turkey. Is there an influence of cultural expectations that are resulting in these findings?

**Response to Reviewer #2:** There may be cultural reasons behind the reasons for violence reported by healthcare professionals, but in this study, when the records of victims of violence were examined, no cultural records were found among the reasons for violence. This interesting reason could be a different research topic.

You also state that the study will make ‘important contributions to the development of prevention policies by evaluating judicial proceedings of reported violence suffered’- **I don’t believe this argument can be made so strongly.**

**Response to Reviewer #2:** Edited in the strengths section according to the reviewer's comment.

In your study you have identified the causes of violence and some of the consequences, which could provide insight when educating current and future health professionals. I think you need to clearly identify how your findings can affect policies and it would be good to have more recommendations emerging from your findings.

**Response to Reviewer #2:** Have been explained and added in the conclusion section.

With the above changes, I believe your article will be stronger and ready for publication.

Thank you.

6. PLOS authors have the option to publish the peer review history of their article (what does this mean?). If published, this will include your full peer review and any attached files.

If you choose “no”, your identity will remain anonymous but your review may still be made public.

Do you want your identity to be public for this peer review? For information about this choice, including consent withdrawal, please see our Privacy Policy.

Reviewer #1: No

Reviewer #2: No

**Data Availability Statement**

Due to research ethics committee restrictions, we are unable to make the data publicly available due to restrictions in the approved protocol. Although no personally identifiable data were collected and the participants' consent form did not inform the participants that their data would be shared outside the research team, even if their identity information was removed. For more information, please contact Dicle University Ethics Committee at [kuruletikdiyar@gmail.com](mailto:kuruletikdiyar@gmail.com).
